# Fabrication of Au–Pd bimetallic dendrites and their catalytic application for 4-nitrophenol reduction

**DOI:** 10.55730/1300-0527.3721

**Published:** 2024-12-24

**Authors:** Jun LIU, Xuanyan LIU, Hongguang LIAO

**Affiliations:** Department of Pharmaceutical and Biological Engineering, Hunan Chemical Vocational Technology College, Zhuzhou, P.R. China

**Keywords:** Heterogeneous catalyst, AuPd bimetallic dendrites, agglomerated structure, catalytic reduction, 4-nitrophenol

## Abstract

Bimetal nanocatalysts hold significant application potential in catalytic reactions due to their compositions, morphologies, and electronic structures. In the present work, a series of AuPd alloy dendrites were successfully prepared using sodium formate, without surfactants or templates. A systematic investigation was then conducted to analyze the composition of the AuPd bimetallic dendrites and the effects of preparation conditions on their structure and catalytic performance. The AuPd alloy dendrites were characterized through various techniques, including scanning electron microscopy, transmission electron microscopy, X-ray energy scattering spectroscopy, X-ray diffraction, and X-ray photoelectron spectroscopy. The results showed that the Au:Pd ratio in the AuPd alloy dendrites closely matched the initial feeding ratio. The catalytic activity and stability of the AuPd alloy dendrites towards 4-nitrophenol reduction were remarkably higher compared to those of the single metal Au and Pd nanocatalysts. Specifically, the Au_1_Pd_1_ alloy dendrites exhibited the highest catalytic reduction activity for 4-nitrophenol, with a reaction rate constant of 1.05 × 10^−2^. Moreover, the stability of the Au_1_Pd_1_ alloy dendrites was also examined. The results demonstrated that the catalytic stability of the Au_1_Pd_1_ alloy dendrites did not experience a significant decrease even after five cycles of operation.

## 1. Introduction

It is well-known that contaminants such as wastewater, exhaust gases, and residues containing 4-nitrophenol (4-NP) are harmful to both humans and the environment [1,2]. 4-Aminophenol (4-AP), a reduction product of 4-NP, has numerous industrial applications and serves as an important intermediate in drug production [3]. Additionally, 4-AP is used in the preparation of various analgesics, antipyretics, photographic developers, stains, corrosion inhibitors, among others [4–6]. Several methods are currently employed for the removal of 4-NP, such as biodegradation [7], microbial degradation [8], photocatalytic degradation [9], and catalytic reduction [10–20]. Among these, catalytic reduction of 4-NP is the most effective, environmentally friendly, and economical method for removing 4-NP [10–20].

Nanocatalysts composed of two different metals are becoming a cutting-edge topic in the cross-disciplinary fields of inorganic chemistry, nanoscience, and catalytic chemistry due to their excellent catalytic properties [10–14,16–17]. Because bimetallic materials share a common energy band between their two components, their electronic structure and surface area can be continuously tuned by adjusting the alloy composition. The adsorption capacity of nanoparticles for reactants and products can be modified by adjusting the distance between the metal’s d-band position and the Fermi level, which, in turn, may affact the catalyst’s activity, selectivity, and stability. Studies have proved that the activity, selectivity and stability of bimetal nanocatalysts depend on their composition, particle size, and nanostructure [10–14,17]. Among these, AuPd micro-/nanomaterials with three-dimensional structures are considered excellent candidates for the catalytic applications in reduction of 4-NP due to their specific morphology, surface area, and composition [10–14,17]. In addition, Pd catalysts are prone to poisoning and have poor stability, which limits their application. The introduction of Au as a co-catalyst in Pd catalysts can significantly improve their anti-poisoning ability and stability by forming Pd-based alloys or core-shell structures [10–11,13,17]. As far as our knowledge extends, a significant portion of AuPd nanocatalysts are nanoparticles of irregular morphology. In the recent decades, a number of groups have reported AuPd catalysts with a wide range of shapes. These include nanoparticles [10–11,13,14], core-shell nanoplates [12], 3D nanoflowers [17], nanowheels [21], nanotetrapods [22], nanowires [23], tripods [24], hollow flowers [25], wheat-like heterostructures [26], nanodroplets [27], hexoctahedra [28], among others. Dendrites are a type of fractal structure consisting of main stems and side branches. In recent years, nanodendrites have been widely used in many fields due to their special structure and high-index crystal faces [28]. To date, several techniques have been used for the preparation of metal dendrites, such as electrochemical deposition [29], galvanic replacement reaction [30,31], dealloying [32], hydrothermal synthesis [33] and ultrasonically assisted templated synthesis [34]. However, reports on the synthesis of AuPd dendrites remain limited [29,32,35,36]. In most available methods for synthesizing AuPd dendritic structures, surfactants or multistep processes may be indispensable [32,35,36].

To the best of our knowledge, the methods for preparing AuPd dendrites mainly include electrochemical deposition [29], dealloying [32], and hydrothermal synthesis [35]. However, these preparation methods are time-consuming. In our previous work, we successfully synthesized AuPd and AgPd dendrites using a one-step electrodeposition process [29]. In this study, we present a method for preparing AuPd bimetallic dendrites using sodium formate, without the need for surfactants or templates. The prepared AuPd bimetallic dendrites exhibited effective catalytic performance and stability for the reduction of 4-NP at room temperature. In addition, these AuPd bimetallic dendrites provide convenience in handling, reuse, and recovery.

## 2. Experimental section

### 2.1. Materials

Chloroauric acid (99.99%), palladium nitrate (99.99%), sodium formate (w% ≥ 99.5, Shanghai, China), sodium borohydride (w% ≥ 98.0), and 4-nitrophenol (w% ≥ 99.0)—all obtained from Sigma Aldrich, Shanghai, China—were used as received without further purification. Deionized water was used to prepare aqueous solutions.

### 2.2. Preparation of AuPd bimetallic dendrites

The uniform AuPd bimetallic dendrites were synthesized by chemical reduction, as described in one of our previous work [37]. In a typical synthesis, multiple mixed solutions were prepared by combining x mL HAuCl_4_ solution (2.43 × 10^−2^ mol/L, x = 4.00, 3.50, 2.50, 1.50, 0.00) and y mL Pd(NO_3_)_2_ solution (3.85 × 10^−2^ mol/L, y = 0.00, 0.74, 1.58, 2.84, 2.52) were prepared. Next, 10 mL NaCOOH solution (1.45 mol/L) was added to each mixed solution. The solutions were then kept stationary for 90 min at room temperature until no further precipitation occurred in each mixed solution. Subsequently, these precipitates were filtered, washed alternately with deionized water and ethanol for 5 times, and dried in a vacuum oven at 60 °C for 4 h. The resulting products were denoted as Au, Au_3_Pd_1_, Au_1_Pd_1_, Au_1_Pd_3_, and Pd catalysts, respectively.

### 2.3. Characterizations of AuPd bimetallic catalysts

The morphology and structure of the prepared AuPd bimetallic catalysts were characterized using cold field Emission scanning electron microscopy (SEM, Hitachi, S-4800, Japan) and high-resolution transmission electron microscopy (TEM, JEOL–1230, Japan). Energy-dispersive X-ray spectroscopy (EDS, HORIBA 7593-H, Japan), X-ray photoelectron spectroscopy (XPS, Quantum 2000, Physical Electronics, USA), and X-ray diffraction (XRD, Bruker, Germany) were used to analyze the chemical composition of the AuPd bimetallic catalysts.

### 2.4. Catalytic reduction activity of AuPd catalyst for 4-NP

To evaluate the catalytic reaction performance of AuPd bimetallic dendrites, a mixed solution was prepared by adding 50 mL of 4-NP (with a concentration of 1 × 10^−4^ mol/L) and 5 mL of NaBH_4_ (with a concentration of 0.2 mol/L). Next, 5 mg of the as-prepared catalyst was added into the mixed solution while stirring at room temperature (approximately 25 °C). Subsequently, at predetermined time intervals, 2.5 mL of the mixed solution was withdrawn from the beaker, and its ultraviolet-visible (UV-vis) absorption spectrum was recorded to monitor changes in the absorption peak of 4-NP during the catalytic reaction. These steps were repeated to evaluate the catalytic performance of each catalyst. The Au_1_Pd_1_ bimetallic dendrites were used repeatedly after being washed with Milli-Q water and then dried under vacuum at 60 °C for 4 h.

## 3. Results and discussion

### 3.1. Characterizations of the agglomerated AuPd bimetallic dendrites

**Figure 1:**
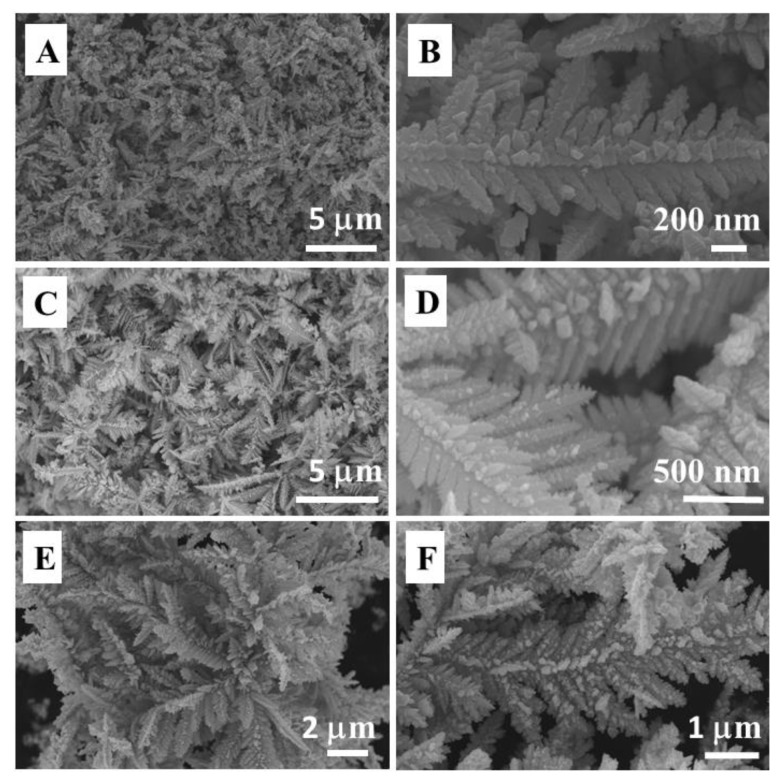
SEM images of bimetallic dendrites for (A,B) Au_3_Pd_1_, (C,D) Au_1_Pd_1_, and (E,F) Au_1_Pd_3_.

AuPd bimetallic samples were prepared via a facile chemical reduction method by using HAuCl_4_ and Pd(NO_3_)_2_ as metal sources and sodium formate as the reductant without any template or surfactant at room temperature for 90 min. Hereafter, the AuPd bimetallic samples prepared from the aqueous solutions of HAuCl_4_/Pd(NO_3_)_2_ mixtures in molar ratios of 1/3, 1/1, and 3/1 will be referred to as Au_1_Pd_3_, Au_1_Pd_1_ and Au_3_Pd_1_, respectively. Figure 1 presents representative SEM images of the Au_3_Pd_1_, Au_1_Pd_1_ and Au_1_Pd_3_ bimetallic samples, demonstrating that the synthesized products exhibit dendritic structures. The Au_3_Pd_1_ and Au_1_Pd_1_ samples exhibit similar dendritic morphologies, as shown in Figures 1A–1D). The dendrite backbone has a total length of approximately 5 μm, with highly symmetrical secondary branches measuring 300–400 nm. The morphology of the Au_1_Pd_3_ sample is shown in Figures 1E–1F. The dendrite structure appears bush-like, with a backbone length of 8–10 μm.

**Figure 2:**
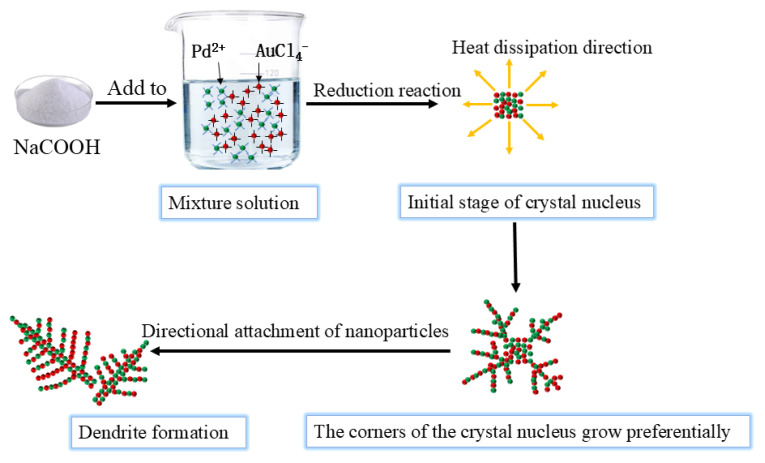
Schematic diagram of the preparation of AuPd bimetallic dendrites by NaCOOH reduction method.

The growth of AuPd bimetallic dendrites is a stepwise and gradual process, as illustrated in Figure 2. Initially, with the addition of NaCOOH, the metal ions in the mixed solution are reduced to form crystal nuclei. In the early stage of crystal nucleation, preferential growth occurs at the corners of the crystal nuclei due to a negative temperature gradient and superior heat dissipation at these sites. Metal atoms then aggregate on the surface of the crystal nuclei, leading to the formation of primary axes, which subsequently give rise to secondary axes. The growth proceeds in either an isotropic or anisotropic manner, ultimately resulting in the formation of nanodendrites with unique morphologies. According to the epitaxial growth theory, the surface energy of Pd is higher than that of Au [38]. Therefore, the growth mode of Pd deposition on Au is similar to island growth, which eventually forms an alloy structure. Meanwhile, the concentration of Pd solution decreases with increasing reaction time; consequently, the growth process may be limited or controlled by the surface reaction. In addition, the different ratios of precursor metals directly affect the morphology and structure of AuPd bimetallic catalyst, as variations in precursor ratios can lead to differences in the reduction rate. Therefore, the morphology and structure of AuPd dendrites can be controlled by adjusting the amount of precursor metal. External factors may also play an important role in the preparation process. The influence of temperature, time, and metal precursor concentration will be studied in the next step.

EDS elemental analysis further confirmed the composition of AuPd dendrites. The results of EDS analysis are listed in Table 1. For comparison, the contents of Au and Pd in the feeding solutions are also shown in Table 1. The results show that the Au contents in the AuPd catalyst is higher than those in the mixed solutions. As far as we know, the standard electrode potential of the AuCl_4_^−^/Au pair is +0.990V (versus the standard hydrogen electrode, SHE) [39,40], and the standard electrode potential of the Pd^2+^/Pd pair is + 0.915V (versus the standard hydrogen electrode, SHE) [40]. The possible reason is that Au is more easily reduced by sodium formate.

**Figure 3:**
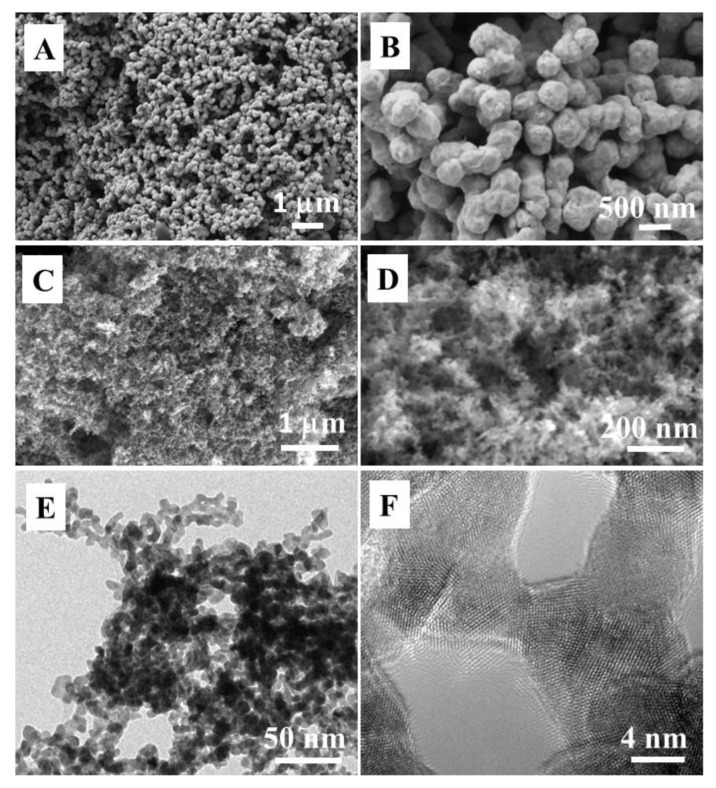
SEM images of the monometallic samples: (A,B) Au, and (C,D) Pd. (E,F) TEM and HR-TEM images of monometallic samples of Pd. The reaction time was 1.5 h.

For comparison, we also studied the monometallic Au and Pd counterparts prepared in the same system. Figure 3A shows the SEM image of the Au sample. It is found that a large number of Au nanoparticles with regular morphology are cross-linked to form a porous structure. The high-magnification SEM image of the Au sample in Figure 3B shows that the size of the Au nanoparticles is about 500 nm, with most of the nanoparticles fused together. Figures 3C and 3D show the SEM images of the Pd sample. The results reveal that the Pd nanoparticles are very fine, and many of them form a porous sponge-like structure.. TEM results of the Pd nanocatalyst further show that the diameter of the Pd nanoparticles is about 10 nm (Figures 3E and 3F). A large number of Pd nanoparticles aggregate together to form a three-dimensional porous, as no protective agent was used in the preparation. The SEM and TEM investigation further confirm that the monometallic Au and Pd catalysts exhibit agglomerated particles, which show significant change in morphology compared to that of AuPd bimetallic dendrites.

**Figure 4:**
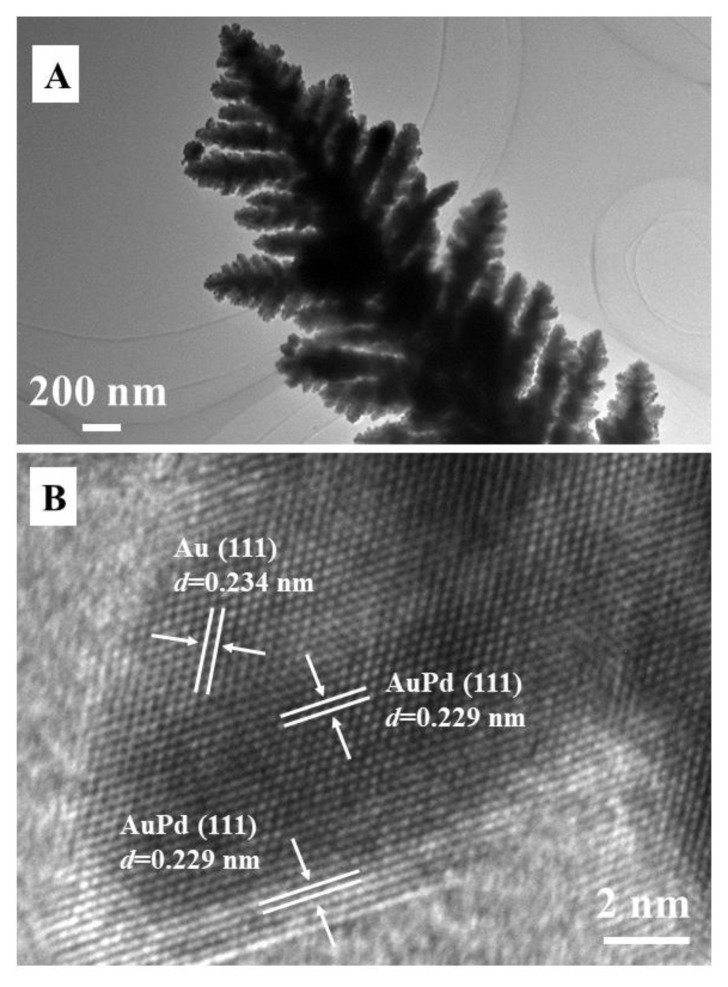
(A) TEM and (B) HRTEM images of the Au_1_Pd_1_ dendrites. The reaction time was 1.5 h.

The morphology and microstructure of a single Au_1_Pd_1_ dendrite were investigated by TEM. Figure 4A shows that the dendrite has a distinct trunk and symmetrical side branches, which are consistent with the SEM results (Figures 1C and 1D). Furthermore, we observed that these branches are composed of small nanoparticles. The HRTEM image of one nanoparticle (Figure 4B) shows a crystalline structure with a lattice spacing of 0.234 nm, which can be attributed to the Au (111) diffraction planes [14,18]. Since the lattice distance of 0.229 nm corresponds to AuPd (111) lattice space [26,32], it can be deduced that the AuPd dendrites are an alloy of Au and Pd.

**Figure 5:**
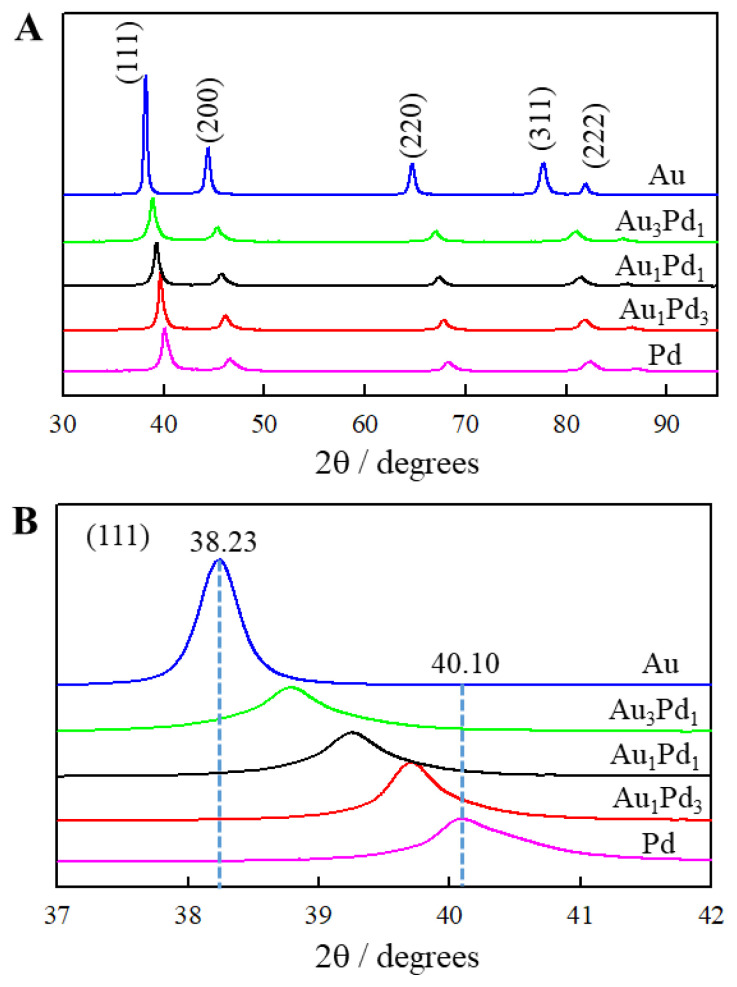
(A) XRD patterns and (B) the corresponding enlarged patterns of (111) diffraction peaks comparison.

XRD is a technique used to analyze the crystal structure of nanomaterials. The XRD patterns of single metal Au, Pd, and AuPd bimetal dendrites are shown in Figure 5. X-ray diffraction peaks can be attributed to (111), (200), (220), (311), and (222) planes of the fcc structure of metallic Pd and/or Au [17,32]. A careful comparison of AuPd, Au, and Pd samples shows that the diffraction peak positions of AuPd dendrites shift towards pure Pd with the increase of Pd content. Figure 4B is an enlarged image of the (111) diffraction peaks, where the peaks of the Au_3_Pd_1_, Au_1_Pd_1_, and Au_1_Pd_3_ dendrites can be observed to fall between the (111) peaks of pure Au and Pd. The XRD results confirm that the AuPd bimetal dendrites are alloys [25,40]. The lattice spacings (d) of the AuPd dendrites, pure Pd and pure Au samples are presented in Table 2.

**Figure 6:**
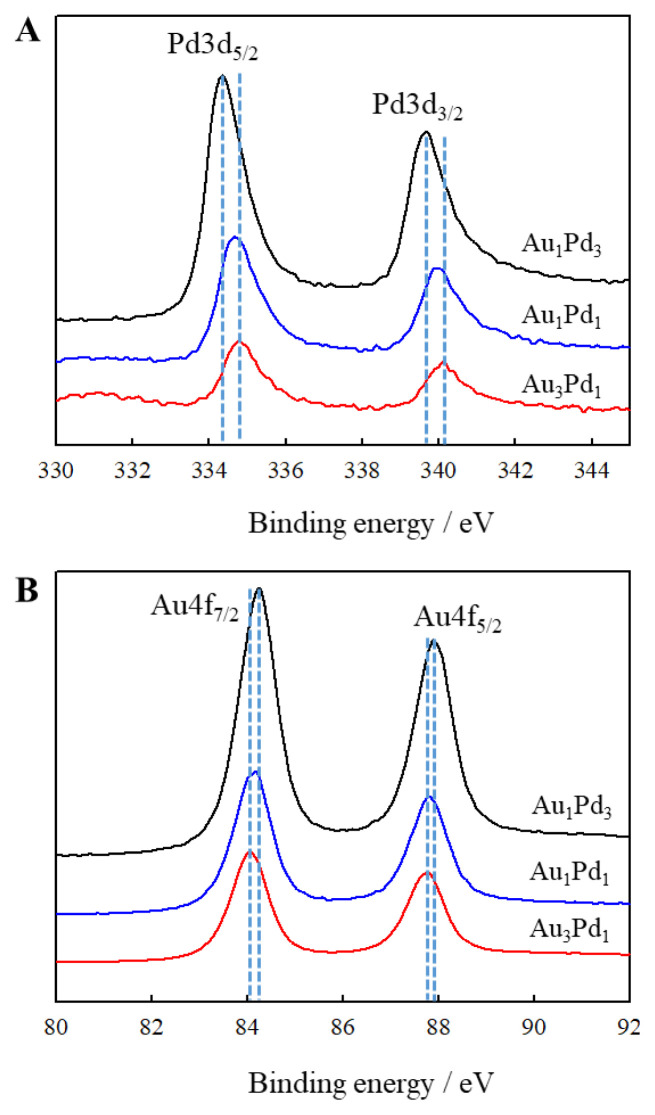
XPS spectra of the AuPd bimetallic dendrites.

XPS spectroscopy was used to study the oxidation states of the elements and the surface compositions of the AuPd bimetallic dendrites. Herein, the peaks at about 334.5 and 339.9 eV are assigned to the binding energies (BEs) of Pd 3d_5/2_ and Pd 3d_3/2_ (Figure 6A), respectively, indicating the presence of Pd in the sample. The 4f orbital electron binding energy of Au has a specific position in the XPS pattern. The peaks at 84.1 and 87.7 eV correspond to the characteristic peaks of Au 4f_7/2_ and 4f_5/2_ (Figure 6B), indicating the presence of Au in the sample. No other oxidation states of Pd and Au were detected in the AuPd bimetallic dendrites, which is consistent with previous literature [22,40]. The BEs of the Pd and Au in AuPd dendrites are summarized in Table 3 in comparison with standard binding energy^1^. It can be observed that with the increase of Pd content in the AuPd sample, the 4f binding energy of Au shifts towards higher binding energies, indicating that there is a charge transfer from Au to Pd in the AuPd bimetallic dendrite. The shift of binding energy can prove the interaction between Au and Pd and form a uniform alloy. The changes in the binding energies of core electrons also indicate the existence of electronic interaction between Au and Pd [22,40], which leads to a transformation in the bulk charge around the atomic sites of AuPd catalysts. The XPS results show that the AuPd dendrite is alloy nanomaterial, consistent with previously reported findings in the literature [22,25,40].

The surface composition of the AuPd samples was analyzed using XPS, with the atomic ratios shown in Table 1. The results show that the atomic percentage of Au on the surface of the AuPd catalysts is slightly higher than that observed in the EDS results. Since the surface energy of Au (1.63 J/m^2^) is lower than that of Pd (2.05 J/m^2^) [41], Au atoms tend to accumulate on the surface of the alloy in order to reduce the energy of the entire system from a thermodynamic perspective.

### 3.2. Catalytic activities of Au–Pd catalysts for the reduction of 4-NP

**Figure 7:**
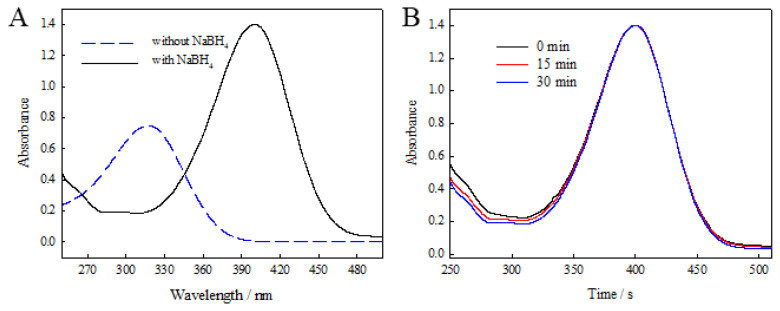
UV-vis spectra of (A) 4-NP with and without NaBH_4_ solution, (B) 4-NP with NaBH_4_ solution.

The catalytic reaction process of 4-nitrophenol was monitored using UV-Vis spectrophotometry. The 4-nitrophenol solution has a strong absorption peak at 317 nm. The absorption peak of 4-nitrophenol showed a clear red shift from 317 nm to 400 nm when sodium borohydride was added to 4-NP solution (Figure 7A), due to the formation of 4-NP salt ions. Figure 7B shows the UV-Vis spectrum of the reaction in the presence of NaBH_4_ for 30 min. The absorption peak at 400 nm remains unchanged, which shows that 4-NP is reduced very slowly by NaBH_4_ in the absence of any catalyst.

**Figure 8:**
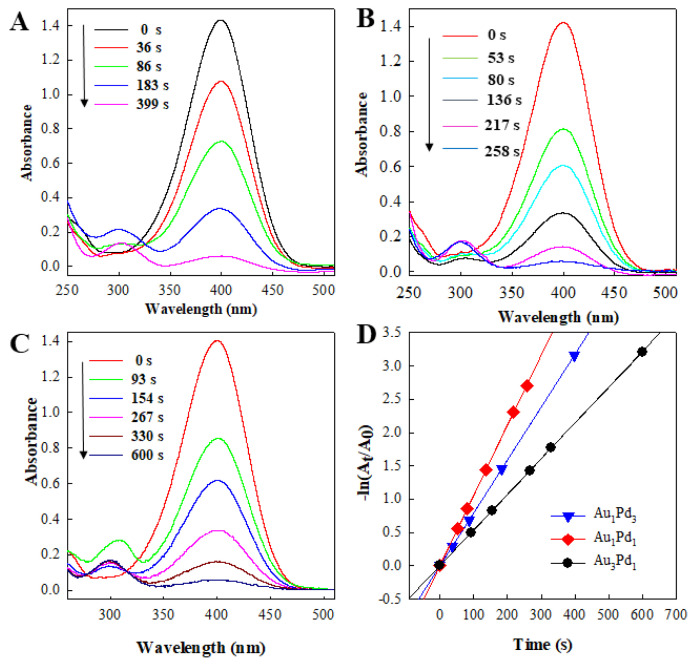
Typical time-dependent evolutions of UV/Vis spectra during the reduction of 4-NP by NaBH_4_ catalyzed by bimetallic dendrites of (A) Au_1_Pd_3_, (B) Au_1_Pd_1_, (C) Au_3_Pd_1_, and (D) the plot of -ln(A_t_/A_0_) versus reaction time for the AuPd catalysts. Reaction parameters: [4-NP] = 1 × 10^−4^ mol/L, 50 mL; [NaBH_4_] = 0.2 mol/L, 5 mL; catalyst mass = 5 mg.

In order to investigate the catalytic activities of the products, we used the as-prepared AuPd products and pure Au and Pd nanoparticles as catalysts for the reduction of 4-NP by sodium borohydride at room temperature, respectively. The 4-NP solution is initially yellow, and its color gradually becomes lighter as the reduction reaction proceeds and finally becomes colorless. Figures 8A–8C show the UV-Vis absorption spectra during the catalytic reduction of 4-NP by different AuPd catalysts. After adding the catalyst, the absorbance at the 400 nm peak gradually decreases, while a new absorption peak appears at 300 nm, which is the characteristic peak of the reduction product 4-AP. The complete conversion of 4-nitrophenol took only 399 s, 258 s, and 600 s for the Au_1_Pd_3_, Au_1_Pd_1_, and Au_3_Pd_1_ catalysts, respectively. In comparison with the Au_1_Pd_3_ and Au_3_Pd_1_ catalysts, it is clear that the Au_1_Pd_1_ catalyst exhibited the highest catalytic performance. The higher catalytic property of Au_1_Pd_1_ could be attributed to the electronic coupling between Pd and Au metals, which is optimized at an atomic percentage of Pd of approximately 45.93%. On the other hand, chemisorption on the catalyst surface varies with the kinds of metals, and the d-band center plays an important role in controlling the chemisorption. The binding energy of the metal-BH_4_^−^ anions and/or 4-nitrophenolate ions in the coupling matrix changes with the composition of the AuPd catalyst. If the adsorption is too strong, it may lead to excessive accumulation of BH_4_^−^ anions and/or 4-nitrophenolate ions on the surface of the catalyst, hindering the diffusion of the reaction substrate and product, thereby reducing the catalytic efficiency. On the other hand, if the adsorption is too weak, the interaction between these ions and the surface of the catalyst becomes insufficient, making it difficult to effectively carry out the electron transfer and chemical bond formation and/or cleavage, which is also not conducive to the catalytic reduction of 4-NP. Thus, AuPd nanocatalyst is expected to achieve the best catalytic activity when the adsorption and desorption energy of BH_4_^−^ anions and/or 4-nitrophenolate ions reach an optimal balance in theory. Therefore, the improved catalytic performance of Au_1_Pd_1_ can be attributed to its special composition, dendritic morphology, and surface electronic structure.

The catalytic reduction of 4-NP is a first-order kinetic reaction when NaBH_4_ is excessive. The relationship between its concentration and rate constant conforms to the first-order kinetic equation: ln (C_t_ /C_0_) = ln (A_t_ /A_0_) = -k_app_ t, where k_app_ is the rate constant, C_0_ and C_t_ are the concentrations of 4-NP at times 0 and t, respectively. A_0_ and A_t_ are absorbance of 4-NP at times t_0_ and t at 400 nm, respectively. The rate constant k_app_ can be obtained by the slope of the linear fitting [11,14,40], as shown in Figure 8D. The k_app_ values for the reduction of 4-NP by the AuPd catalysts are calculated to be 7.92 × 10^−3^ s^−1^, 10.5 × 10^−3^ s^−1^, 5.36 × 10^−3^ s^−1^ for Au_1_Pd_3_, Au_1_Pd_1_, and Au_3_Pd_1_, respectively. Therefore, the catalytic efficiency for AuPd catalysts follows the order: Au_1_Pd_1_ > Au_1_Pd_3_ > Au_3_Pd_1_. The real surface areas of the Au_1_Pd_1_, Au_1_Pd_3_, and Au_3_Pd_1_ catalysts were determined using double-layer capacitance measurements, yielding values of 43.7 m^2^ g^−1^, 51.2 m^2^ g^−1^, and 48.3 m^2^ g^−1^, respectively [42]. Moreover, the turnover frequency (TOF) values of the catalysts are presented in Table 4. These results indicate that the AuPd catalysts exhibit outstanding catalytic activity for 4-NP reduction.

**Figure 9:**
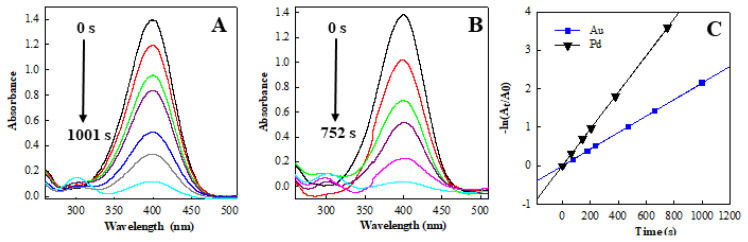
Typical time-dependent evolutions of UV/Vis spectra during the reduction of 4-NP by NaBH_4_ catalyzed by catalysts of (A) Au, (B) Pd are shown, and (C) the plot of ln(At/A0) versus reaction time for the Au and Pd catalysts is also presented. Reaction parameters: [4-NP] = 1 × 10^−4^ mol/L, 50 mL; [NaBH_4_] = 0.2 mol/L, 5 mL; catalyst mass = 5 mg.

In this study, the catalytic activities of Au and Pd nanoparticles were also examined, and the results are presented in Figure 9. The catalytic reaction of 4-NP is completed within 1001 s using the Au nanoparticles as the catalyst (Figure 9A), which is longer than that of the as-prepared Pd nanoparticles (752 s, Figure 9B). Figure 9C illustrates the linear relationships between reaction time and -ln(A_t_/A_0_). The k_app_ values of catalytic reduction reaction for 4-NP by Au and Pd nanoparticles were calculated to be 2.15 × 10^−3^ s^−1^ and 4.76 × 10^−3^ s^−1^, respectively.

**Figure 10:**
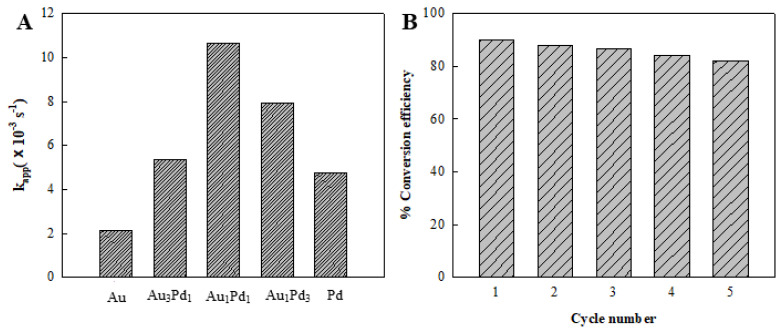
(A) The k_app_ values of AuPd, Au, and Pd catalysts are presented. (B) The reusability of the Au_1_Pd_1_ as a catalyst for the reduction of 4-NP with NaBH_4_ is investigated. Reaction parameters: [4-NP] = 1 × 10^−4^ mol/L, 50 mL; [NaBH_4_] = 0.2 mol/L, 5 mL; mass of fresh Au_1_Pd_1_ catalyst = 5 mg.

The k_app_ values of these catalysts are displayed in Figure 10A, suggesting that the order of the catalytic activities of AuPd bimetallic dendrites, as well as single-component Au and Pd catalysts, follows Au_1_Pd_1_ > Au_1_Pd_3_ > Au_3_Pd_1_ > Pd > Au. As far as we know, the catalytic process of 4-NP includes the following steps: 4-nitrophenolate ions and BH_4_^−^ anions are adsorbed on the surface of AuPd catalysts; AuPd alloy initiates the electron transfer between BH_4_^−^ anions and 4-nitrophenolate ions, that is, the catalyst receives the electron pair separated by BH_4_^−^ anions and transfers it to 4-nitrophenolate ions. Herein, AuPd catalysts play a crucial role in the hydrogenation of 4-NP to 4-AP. Notably, AuPd catalysts demonstrate superior catalytic activity compared to single Au and Pd catalysts. Furthermore, another possible reason is that the electronic structure of Pd changes when Au and Pd form the alloy, which enhances the hydrogen adsorption at unsaturated active sites. Ion adsorption on the catalyst surface facilitates overcoming the kinetic barriers, thereby accelerating the catalytic reduction of 4-NP.

Catalyst recyclability is a crucial factor in evaluating catalytic activity. The stability of Au_1_Pd_1_ was tested over five successive cycles (Figure 10B). After each cycle, the Au_1_Pd_1_ catalyst was collected by filtration, washed five times with Milli-Q water, dried under vacuum at 60 °C for 4 h, and reused in the next cycle. More than 82% of the 4-NP was converted to 4-AP after 5 cycles. The catalytic activity of the Au_1_Pd_1_ catalyst showed a slight decline from the first cycle to the fifth cycle. A minor loss of catalyst occurred during filtration and separation prior to the use of next cycle. In addition, catalytic performance was affected by a series of factors such as morphology, structure, surface composition, and the interaction between metal nanoparticles.

**Figure 11:**
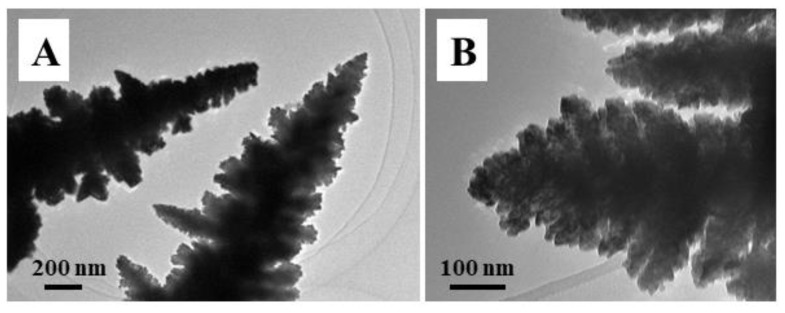
TEM images of the bimetallic dendrites of Au_1_Pd_1_ after the 5th cycle for the reduction of 4-NP.

Figure 11 shows the TEM image of the Au_1_Pd_1_ catalyst after the fifth run of the reproducibility. By comparing the newly prepared Au_1_Pd_1_ bimetallic dendrites (Figure 4A), subtle changes in the morphology of the AuPd nanodendrites can be observed after five cycles. Some nanoparticles within the nanodendrites have undergone fusion. Furthermore, the surface areas of the AuPd catalysts were also evaluated after multiple cycles of use. The real surface areas of the Au_1_Pd_1_, Au_1_Pd_3_, Au_3_Pd_1_ catalysts were estimated from the double layer capacity measurement, yielding values of 42.4 m^2^ g^−1^, 49.6 m^2^ g^−1^, and 47.7 m^2^ g^−1^, respectively. This might be due to the agglomeration of some nanoparticles, which led to a reduction in surface area and might be the main reason for the decrease in their catalytic activities.

Furthermore, the AuPd catalysts after five catalytic reduction cycles were characterized by XPS (Figure S). The surface composition is presented in Table 1. Compared with the newly prepared AuPd catalysts, the Pd content on the surface of the AuPd catalysts after multiple cycles is slightly decreased, resulting in a reduction in the number of active Pd cites on the catalyst surface. This may be a key reason for the slight decrease in catalytic activity. Further analysis of the fine structure in the XPS spectrum reveals a specific electronic interaction between Au and Pd. This interaction may be one of the key factors contributing to the high catalytic activity exhibited by this catalyst.

## 4. Conclusion

AuPd nanodendrites in an aggregated state were prepared using a one-step displacement method using NaCOOH as the reducing agent, under aqueous conditions and at room temperature. The morphology and structure of these nanodendrites vary depending on the composition of AuPd. In addition, the catalytic activities of AuPd, as well as single-metal Pd and Au nanocatalysts, were compared in the catalytic degradation of 4-NP at room temperature. The results showed that the order of catalytic activity was Au_1_Pd_1_ > Au_1_Pd_3_ > Au_3_Pd_1_ > Pd > Au. The recycling performance of the Au_1_Pd_1_ nanocatalyst was also investigated. Its catalytic activity remained stable after five recycling cycles, indicating good stability. Compared with previous literature studies, the innovation of this research lies in the fact that the prepared AuPd alloy catalyst has an aggregated dendritic structure, which not only contributes to the variation in catalytic performance with different compositions but also endows the catalyst with the advantage of easy separation from the reaction system. It has potential application prospects in the field of catalysis and is expected to provide new ideas and directions for future related catalytic research and industrial applications.

## Supporting information

Figure S XPS spectra of the AuPd bimetallic dendrites after the 5th cycle for the reduction of 4-NP.

## Figures and Tables

**Table 1:** Content of Pd and Au in AuPd dendrites and feeding solutions.

Sample	at.%						
	Au*	Pd*	Au^	Pd^	Au#	Pd#	Pd&
Au_1_Pd_3_	27.03	72.97	28.49	71.51	27.98	72.02	75.00
Au_1_Pd_1_	53.26	46.74	54.03	45.97	54.07	45.93	50.00
Au_3_Pd_1_	79.12	20.88	80.08	19.92	80.24	19.76	25.00

Au*, Pd*: EDS analysis.

Au^, Pd^: XPS analysis after 5th cycle for the reduction of 4-NP.

Au#, Pd#: XPS analysis.

Pd &: Pd in feeding solutions.

**Table 2:** Lattice spacings (*d*) of AuPd dendrites and pure Pd, pure Au samples.

Sample	*d*_(111)_ /nm	*d*_(200)_ / nm	*d*_(220)_ / nm	*d*_(311)_ / nm	*d*_(222)_ / nm
Pd	0.22470	0.19519	0.13720	0.11705	0.11210
Au_1_Pd_3_	0.22730	0.19734	0.13889	0.11849	0.11347
Au_1_Pd_1_	0.22991	0.19949	0.14057	0.11992	0.11484
Au_3_Pd_1_	0.23251	0.20164	0.14225	0.12135	0.11621
Au	0.23512	0.20379	0.14393	0.12278	0.11758

**Table 3:** BEs of Pd and Au in AuPd dendrites.

Sample	BE			
	Pd3*d*_5/2_	Pd3*d*_3/2_	Au*f*_7/2_	Au*f*_5/2_
Au_1_Pd_3_	334.31	339.64	84.24	87.90
Au_1_Pd_1_	334.64	339.93	84.14	87.79
Au_3_Pd_1_	334.80	340.13	84.06	87.76
SBE	335.00	340.30	84.00	87.70

SBE: standard binding energy.

**Table 4:** Comparison of catalytic activity of present AuPd catalyst with relevant reported results.

Catalyst	Molar ratio of NaBH_4_ to 4-NP	K_app_×10^−3^ s^−1^	TOF×h^−1^	T(K)	Reference
Au_1_Pd_1_ catalyst	200:1	10.5	329	298	This work
Au_1_Pd_3_ catalyst	200:1	7.92	227	298	This work
Au_3_Pd_1_ catalyst	200:1	5.36	89	298	This work
Au_20_-Hb NPs	2500:1	3.32	6768	298	[15]
Ag/Cu BTNPs	50:1	331	1800	298	[16]
AuPd NF	75:1	10.9	14.10	298	[17]
Au NPs	98:1	11.8	1090	298	[18]
Ag NPs	98:1	0.4	50	298	[18]
Pd NPs	98:1	18.3	2250	298	[18]
Pd@CCM catalyst	/	9.51	38.4	298	[19]
Pt@Ag NPs	9:1	5.91	412	298	[20]

## Footnotes

1 Wiley (2000). The Elements of Native Oxides [online]. Website http://www.xpsdata.com [accessed 02 October 2000].
