# Supplementary figures and images for: Fabrication of Au–Pd bimetallic dendrites and their catalytic application for 4-nitrophenol reduction
Source: Turk J Chem. 2024 Dec 24;49(2):191–203. doi: 10.55730/1300-0527.3721 (PMC12068668; doi:10.55730/1300-0527.3721)

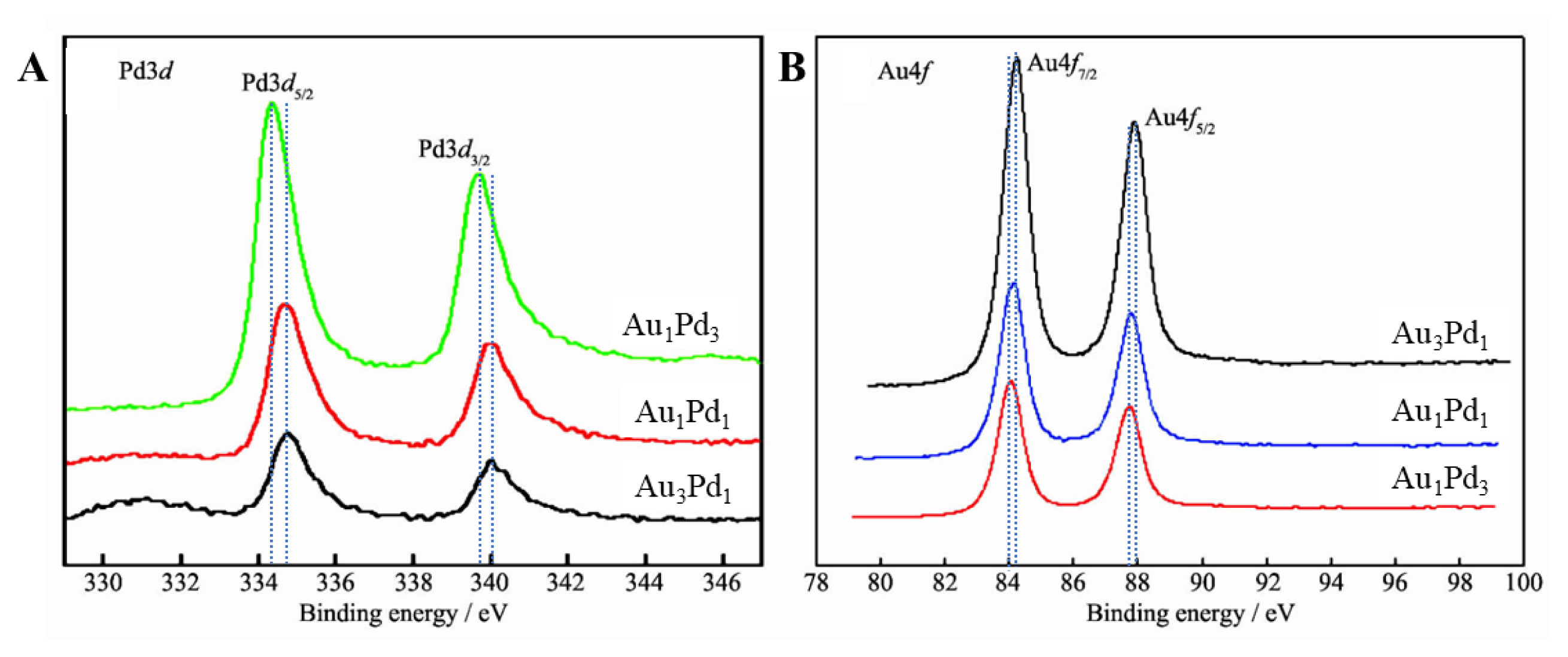

Supplement: Figure S — XPS spectra of the AuPd bimetallic dendrites after the 5th cycle for the reduction of 4-NP. [file tjc-49-02-191s1.tif]
